# Draft Genome Sequence of the Polysaccharide-Degrading Marine Bacterium *Pseudoalteromonas* sp. Strain A601

**DOI:** 10.1128/genomeA.00590-17

**Published:** 2017-07-20

**Authors:** Jungang Li, Yuanyuan Cheng, Dandan Wang, Junge Li, Yanhui Wang, Wenjun Han, Fuchuan Li

**Affiliations:** a School of Life Science and Biotechnology, Mianyang Normal University, Mianyang, China; b National Glycoengineering Research Center, Shandong University, Jinan, China; c Department of Food Science and Engineering, Shandong Agriculture and Engineering University, Jinan, China

## Abstract

*Pseudoalteromonas* sp. strain A601 is a marine bacterium with excellent polysaccharide-degrading capabilities. Here, we present a high-quality draft genome sequence of strain A601 with a size of approximately 4.89 Mb and a mean G+C content of 40.0%. Various putative polysaccharide-degrading genes were found in the draft genome.

## GENOME ANNOUNCEMENT

*Pseudoalteromonas* belongs to the order *Alteromonadales* in the class *Gammaproteobacteria* according to differences in small subunit rRNA gene sequences (1). The *Pseudoalteromonas* strains require seawater environments for growth and are therefore true marine bacteria. Allowing for rare exceptions, *Pseudoalteromonas* strains are associated with healthy animals or algae, and few strains are known as pathogenic or opportunistic (2). To date, 43 *Pseudoalteromonas* genome sequences of different strains have been published, but only 5 genomes are complete.

In the present study, we report the draft genome sequence of *Pseudoalteromonas* sp. strain A601, which was isolated from costal sediment collected from the Jiaozhou Bay of China on 9 March 2015. This strain has been deposited in the China General Microbiological Culture Collection Center (accession no. CGMCC13708). Phylogenetic analysis based on partial 16S rRNA gene sequencing showed that strain A601 (GenBank accession no. KY859766) displayed highest similarity to the type strain *Pseudoalteromonas atlantica* ATCC 19262 (97.61%). Reported *Pseudoalteromonas* strains are organisms of interest in the fields of ecological and pharmaceutical sciences due to their excellent capabilities of forming biofilms and synthesizing bioactive molecules (3, 4). Interestingly, function screening of strain A601 revealed its significant capabilities for enzymatic degradation and bacterial utilization of marine-derived polysaccharides, such as agarose, alginate, and chitin.

Genomic DNA of strain A601 was extracted from cultured cells using an alkaline lysis method (5). Genome sequencing was determined using Illumina/Solexa MiSeq technology at Shanghai Majorbio Bio-pharm Technology Co., Ltd. (Shanghai, China), generating 5,603,212 paired-end reads. Filtered data were assembled by SOAPdenovo version 2.04 (6) to generate scaffolds. Transfer RNA genes (tRNAs) were predicted with tRNAscan-SE version 1.3.1 (7). Ribosomal RNA genes (rRNAs) were predicted with Barrnap software version 0.4.2 (8). Interspersed repeats were predicted using Repeat Masker (http://www.repeatmasker.org). Tandem repeats were analyzed using Tandem Repeat Finder (http://tandem.bu.edu/trf/trf.html). Protein-coding sequences were predicted with Glimmer version 3.02 software (9) using default parameters and annotated using BLAST searches of nonredundant protein sequences from the NCBI, Swiss-Prot/TrEMBL, COG (10), Gene Ontology (11), and KEGG (12) databases. Genes of interest likely to be involved in the breakdown of sulfated algal polysaccharides were manually evaluated.

The assembled genome consisted of 89 scaffolds comprising 4,894,552 bp with a mean G+C content of 39.978%. A total of 4,488 genes were predicted, and 98 RNA genes (4 rRNAs and 94 tRNAs) and 4,390 protein-coding genes were numbered. Putative functions of the majority of the protein-coding genes were prognosticated. In the draft genome of *Pseudoalteromonas* sp. strain A601, we have preliminarily found the following multiple encoding genes of putative polysaccharide-degrading enzymes: eight glucosidases (EC 3.2.1.20 and EC 3.2.1.21), six chitinases (EC 3.2.1.14), five alginate lyases (EC 4.2.2.3 and EC 4.2.2.11), five agarases (EC 3.2.1.81), five glucanases (EC 3.2.1.4, EC 3.2.1.6, EC 3.2.1.39, and EC 3.2.1.176), three amylases (EC 3.2.1.1, EC 3.2.1.2, and EC 3.2.1.3), and one xylanase (EC 3.2.1.8 and EC 3.2.1.37). These results suggest an amazing capability of *Pseudoalteromonas* sp. strain A601 to degrade polysaccharides derived from marine animals and plants.

### Accession number(s).

This whole-genome shotgun project has been deposited at DDBJ/EMBL/GenBank under the accession no. MXQF00000000. The version presented here is the first version, MXQF01000000.
